# Impact of cardiovascular magnetic resonance assessment of ejection fraction on eligibility for implantable cardioverter defibrillators

**DOI:** 10.1186/1532-429X-13-S1-O32

**Published:** 2011-02-02

**Authors:** Subodh B Joshi, Kim A Connelly, Mark Hansen, Sean McSweeney, Jerome Liu, Yuesong Yang, Laura Jimenez-Juan, Abdul Al-Hesayen, Iqwal Mangat, Paul Dorian, Graham A Wright, Andrew Crean, Anish Kirpalani, Andrew T Yan, Howard Leong-Poi

**Affiliations:** 1 St Michael's Hospital, Toronto, ON, Canada; 2 Sunnybrook Health Sciences Centre, Toronto, ON, Canada; 3 Toronto General Hospital, Toronto, ON, Canada

## Objective

To determine whether cardiovascular magnetic resonance (CMR) for left ventricular ejection fraction (LVEF) assessment changes implantable cardioverter defibrillator (ICD) eligibility when compared with echocardiography.

## Background

A markedly reduced LVEF is considered an indication for ICD placement for the primary prevention of sudden cardiac death. However, despite strict LVEF criteria, most guidelines do not specify the technique by which LVEF should be measured.

## Methods

The study population consisted of patients referred for LVEF measurement by CMR, for consideration of ICD implantation, who also underwent echocardiography within 30 days of the CMR. LVEF was assessed on echocardiography using Simpson’s biplane method. LVEF was determined from CMR based on manual planimetry of SSFP cine images of contiguous left ventricular short axis slices. CMR and echocardiography derived LVEFs were reported by two independent blinded observers.

## Results

Forty-nine (49) eligible patients were identified (10 female, mean age 61 +/- 15 years, 24 with ischemic etiology) who underwent CMR between March 20, 2007 and Aug 12, 2010. The median number of days between CMR and echo was 3 (IQR 1 to 10 days). The mean LVEF by CMR and echo was 31 +/- 15 %, and 34 +/- 15%, respectively, (p =0.009), with a correlation coefficient (r) between the two of 0.86. Using Bland Altman analysis, the mean difference (CMR - echo) was - 3.1 % with limits of agreement of - 18 to 12 %. CMR resulted in reclassification regarding ICD eligibility in 10 (20 %) patients using an LVEF threshold of 35 %, and 8 (16 %) using an LVEF threshold of 30 %. Tables 1 and 2.

**Table 1:** LVEF Threshold 30 %

	Echo LVEF >=30%	Echo LVEF <30%	Total
CMR LVEF >=30 %	21	1	22
CMR LVEF <30 %	7	20	27
Total	28	21	49
Kappa = 0.68			

**Table 2:** LVEF Threshold 35 %

	Echo LVEF >= 35 %	Echo LVEF < 35%	Total
CMR LVEF >=35%	15	3	18
CMR LVEF <35%	7	24	31
Total	22	27	49
Kappa = 0.58			

## Conclusion

In this cohort of patients being considered for ICD implantation, echocardiography systematically over-estimated LVEF. Using strict LVEF criteria, CMR changed the eligibility for ICD in a substantial proportion of patients, with, in most cases, CMR determining that the patient was ICD eligible when they were not based on echocardiography.

